# Use of simulator-based medical procedural curriculum: the learner's perspectives

**DOI:** 10.1186/1472-6920-10-77

**Published:** 2010-11-08

**Authors:** David Shanks, Roger Y Wong, James M Roberts, Parvathy Nair, Irene WY Ma

**Affiliations:** 1 Department of Medicine, University of British Columbia, Vancouver, BC, Canada; 2 Department of Medicine, University of Calgary, Calgary, AB, Canada

## Abstract

**Background:**

Simulation is increasingly used for teaching medical procedures. The goal of this study was to assess learner preferences for how simulators should be used in a procedural curriculum.

**Methods:**

A 26-item survey was constructed to assess the optimal use of simulators for the teaching of medical procedures in an internal medicine residency curriculum. Survey domains were generated independently by two investigators and validated by an expert panel (n = 7). Final survey items were revised based on pilot survey and distributed to 128 internal medicine residents.

**Results:**

Of the 128 residents surveyed, 106 (83%) responded. Most responders felt that simulators should be used to learn technical skills (94%), refine technical skills (84%), and acquire procedural teaching skills (87%).

Respondents felt that procedures most effectively taught by simulators include: central venous catheterization, thoracentesis, intubation, lumbar puncture, and paracentesis. The majority of learners felt that teaching should be done early in residency (97%).

With regards to course format, 62% of respondents felt that no more than 3-4 learners per simulator and an instructor to learner ratio of 1:3-4 would be acceptable.

The majority felt that the role of instructors should include demonstration of technique (92%), observe learner techniques (92%), teach evidence behind procedural steps (84%) and provide feedback (89%). Commonly cited barriers to procedural teaching were limitations in time, number of instructors and simulators, and lack of realism of some simulators.

**Conclusions:**

Our results suggest that residents value simulator-based procedural teaching in the form of small-group sessions. Simulators should be an integral part of medical procedural education.

## Background

Simulation is increasingly utilized in the education of procedural skills. Simulation offers several potential advantages over traditional methods of medical training. First, simulators allow for learning and practicing of technical skills in a safe and controlled environment, without posing danger to patient well-being [1]. In addition, training on simulators improves technical skills [2-4]. In an era of increasing awareness of medical errors and concern for patient safety [5,6], such opportunities are invaluable. Secondly, simulators are flexible educational tools. For example, the use of simulators in teaching technical skills can range from a bench-top skill station [4,7,8] to a more complex integrated clinical procedural scenario [9]. Finally simulators allow learners the opportunities for deliberate practice [10], an important factor in the acquisition of skills and expertise [11].

Procedural training using simulation has a long history, dating as far back as 600 bc [12], and is increasingly used in medical education. Despite better understanding of how best to incorporate simulation technology into an educational curriculum [10], little information exists on the use of simulators in procedural teaching from the learner's perspective. Although the Accreditation Council for Graduate Medical Education stipulates that internal medicine residency programs must provide residents with access to training using simulation [13], no specific guidance or guidelines exist in terms of how to implement simulation-based education. Consistent with the principles of adult learning theory, a better understanding of the learners' perspective can assist educators in deciding how to implement a simulation curriculum [14].

Given the lack of guidelines on the implementation of simulation-based education, the aim of the current study is to establish learner preferences in simulation-based procedural education in internal medicine. This was done by the generation of a 26-item web-based survey that was distributed to all internal medicine residents at an academic institution.

## Methods

### Study Population

All 128 core internal medicine (PGY-1 to PGY-4) residents at the University of British Columbia were invited to participate and complete a voluntary anonymous on-line survey in 2009. At the University of British Columbia, since 2007, all internal medicine residents were invited to attend 2-3 hour workshops on procedural simulation training on task trainers, in small groups of ranging between two to eight in size. Generally one to two instructors were present per workshop. Procedural training in central venous catheterization had been mandatory since 2008. Training in arterial blood gas sampling, intubation, lumbar puncture, paracentesis, thoracentesis, and knee arthrocentesis was made available as elective workshops.

Our study was approved by the University of British Columbia Behavioural Research Ethics Board and the University of British Columbia Internal Medicine Residency Training Committee.

### Survey Development

Based on review of the literature [10,15,16] and clinical teaching experience, survey domains were generated independently by two investigators (DS, IM). Key domains identified included learner background, experience, motivation and attitude towards simulation; learning environment, course content and format; instructor roles, skills and expertise. Survey items were generated to address topics within each of these domains. Feedback from an expert panel (N = 7) supported content validity of survey domains. Members from the expert panel consisted of three clinician educators, two academic surgeons, one intensivist, and one academic nephrologist. This expert panel rated relative importance of survey items and pilot survey items were modified based on expert panel rating resulting in a 31-item survey. This 31-item survey was then piloted to nine internal medicine trainees: five trainees were recent graduates of the core internal medicine program (PGY-5s) at same institution (University of British Columbia). Four trainees were PGY-2s at other Canadian academic institutions. Survey items were revised based on input from this pilot survey with respect to question phrasing, clarity, flow, redundancy, and ease of use, resulting in a final 26-item survey (additional file 1). Test-retest reliability of the 26-item survey was assessed four weeks after the initial pilot and showed a Kappa score of 0.71 and ρ of 0.88, indicating good to substantial agreement [17]. This 26-item on-line survey, administered using an electronic survey instrument (Survey Monkey, http://www.surveymonkey.com, accessed December 1, 2009) was distributed electronically in February 2009 to all 128 internal medicine residents via a personalized invitation e-mail. Participants were offered the opportunity to enter into a drawing for one of three book prizes in an attempt to maximize the response rate. Reminder e-mails were sent four weeks following the initial invitation.

### Statistical Analysis

Descriptive statistics were presented for baseline characteristics. Test-retest agreement was assessed using Kappa score and Spearman correlation coefficient. Statistical analyses were conducted using Stata version 11.0 (StataCorp LP, College Station, TX) and SAS version 9.1 (SAS Institute, Cary, NC).

## Results

Of the 128 invited residents, 106 completed the survey (83%). Table 1 shows the baseline characteristics of the participants. The majority of the participants had prior experience on simulators, primarily through attending formal procedural simulator courses (88%). The majority of participants (94%) felt that simulators are helpful in the acquisition of procedural skills (Table 1). Fewer participants (66%) felt that simulators are useful in the assessment of procedural skills.

**Table 1:** Characteristics of Participants Who Completed Survey (N = 106)

Characteristic	No. (%)
Sex	

Male	63 (59)

Female	42 (39)

Not reported	1 (0.9)

Level of training	

PGY-1	35 (33)

PGY-2	38 (36)

PGY-3/4	32 (30)

Prior Experience on simulators	

Received prior formal training on simulators	93 (88)

Received prior informal training on simulators	20 (19)

No prior training on simulators	4 (4)

Simulators are useful in	

Acquisition of procedural skills	100 (94)

Learning teaching skills	92 (87)

Refining procedural skills	89 (84)

Assessment of procedural skills	70 (66)

### Course Content and Format

With respect to course content, over 80% of participants would opt to undergo training in the following procedures using simulators: central venous catheterization, ultrasound guided central venous catheterization, thoracentesis, intubation, lumbar puncture, and paracentesis (Table 2). Procedures that 50% or fewer participants would opt to undergo training in included: peripheral intravenous access, peripherally inserted central catheter, and introductory course on sterile technique, suturing, hand-ties, and local anesthetic administration.

**Table 2:** Survey Results of 106 Participants' View on Course Content

Characteristic	No. (%)
Number of participants interested in undergoing procedural training on simulators in	

Central venous catheterization	92 (87)

Ultrasound-guided central venous catheterization	97 (92)

Thoracentesis	95 (90)

Intubation	93 (88)

Lumbar puncture	90 (85)

Paracentesis	89 (84)

Peripherally inserted central catheter	61(38)

Peripheral intravenous	53 (50)

Arterial blood gas sampling	46 (43)

Introductory course on sterile technique, suturing, hand-ties, local anesthetic administration	53 (50)

Number of participants who feel simulation training should be mandatory	92 (87)

Simulator training for these procedures should be made mandatory	

Central venous catheterization	45 (42)

Ultrasound-guided central venous catheterization	42 (40)

Intubation	38 (36)

Lumbar puncture	36 (34)

Arterial blood gas sampling	21 (20)

Thoracentesis	32 (30)

Paracentesis	29 (27)

Incorporating a clinical scenario into procedural teaching using simulators would be very useful or somewhat useful	83 (78)

**Figure 1:**
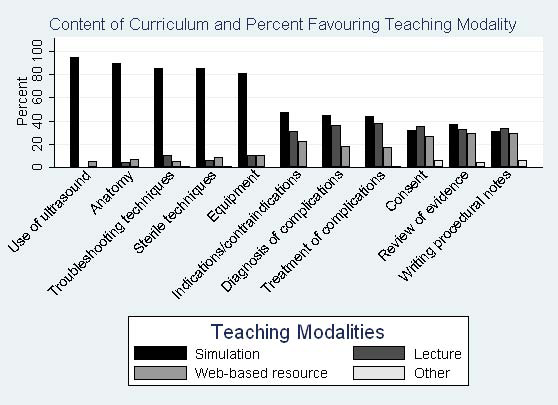
**Content of Curriculum and Percent Favouring Teaching Modality**. Bar graph of percent of participants favouring teaching modality.

Aspects of procedures the participants felt were best taught using simulation included: use of ultrasound, review of anatomy, troubleshooting techniques, sterile techniques, and review of equipment (Figure 1). For remaining aspects of the procedures (review of indications, contraindications, diagnosis and treatment of complications, consent, review of evidence, and procedural notes), participants did not significantly favour the use of simulators over didactic lectures, web-based resources, and other modalities such as bedside teaching and small-group sessions.

Incorporating a clinical scenario into procedural teaching using simulators was felt to be very useful or somewhat useful by 83 of the participants, (Table 2). The majority of the participants (97%) felt that simulation should be offered early in residency (Table 3). Of the 103 participants who felt that simulation should be offered early in residency, 74 (72%) felt that additional sessions should be offered throughout residency. Small group format of ≤ 3 hour duration was favoured by the participants. Although 87% of participants felt that simulator-based procedural training should be made mandatory in a residency program, participants did not agree on which procedure should be made mandatory (Table 2).

**Table 3:** Survey Results of 106 Participants' View on Course Format and Instructor Characteristics

Characteristic	No. (%)
**COURSE FORMAT**	

Location favored	

Center away from hospital	2(2)

Center within hospital	74 (70)

Directly on ward	20 (19)

Doctor's lounge	4 (4)

Other or no opinion	6 (6)

When should simulator courses be offered	

At beginning of residency, with additional sessions throughout residency	74 (70)

At beginning of residency	29 (27)

Later in residency (PGY-2/3)	1(1)

No opinion	2 (2)

Training session times should be specified and protected	74 (70)

Training session times should be specified but unprotected	24 (23)

Simulators should be freely available	67 (63)

Maximum acceptable learner to simulator ratio	

No more than 1-2 learners per simulator	29 (27)

No more than 3-4 learners per simulator	66 (62)

No more than 5-6 learners per simulator	9 (8)

>6 learners per simulator is acceptable	1 (1)

Minimum acceptable instructor to learner ratio	

No fewer than 1 instructor per 1-2 learners	20 (19)

No fewer than 1 instructor per 3-4 learners	68 (64)

No fewer than 1 instructor per 5-6 learners	16 (15)

1 instructor for > 6 learners is acceptable	1 (1)

Optimal duration of a simulator session	

One hour	21 (20)

Two hours	56 (53)

Three hours	20 (19)

>3 hours	2 (2)

Full day session (with breaks)	5 (5)

**INSTRUCTOR CHARACTERISTICS**	

Medical simulator sessions should be taught by	

An attending physician	87 (82)

A senior resident (or fellow)	91 (86)

A trained technician	53 (50)

The instructor should	

Demonstrate technique	98 (92)

Observe my procedure	98 (92)

Teach evidence behind procedural steps	89 (84)

Provide feedback	94 (89)

### Instructor Characteristics

With respect to instructor characteristics, 74 participants (70%) strongly disagreed or somewhat disagreed with the statement that the role of a supervisor in a simulation-based procedural education session is superfluous. The majority of participants felt that simulation-based procedural training should be taught by either an attending (82%) or senior resident (86%). Only 50% felt that a trained technician would be a suitable instructor. Participants strongly agreed or somewhat agreed that the role of an instructor included demonstration of technique (92%), observe learner techniques (92%), teach evidence behind procedural steps (84%) and provide feedback (89%).

### Barriers to Simulation-Based Procedural Education

Lastly, 52 (49%) participants answered an open-ended question regarding perceived barriers to simulation-based procedural education. Time is the most commonly cited barrier to institution of simulation-based procedural education (*n *= 31; 60%), followed by limited availability of simulators (*n *= 12; 23%), realism of simulators (*n *= 12; 23%), number of available procedural teachers (*n *= 10; 19%), and overall cost of program (*n *= 3; 6%).

## Discussion

Simulation is an attractive educational modality. Simulation allows for deliberate practice [18] and allows learner to acquire technical expertise by advancing through Fitts and Posner's three-stage theory of motor skill acquisition: cognition, integration, and automation [19,20]. While educational research has substantially advanced our understanding in terms of what educational elements in simulation best enhances learning [10], considerably less research has addressed simulation from the view point of the learner. Consistent with adult learning theory, where adults are viewed as self-directed learners, a better understanding of the learners' perspectives on education can assist educators in designing and implementing a simulation-based procedural curriculum [21]. In the absence of guidelines on how to implement simulation-based procedural curriculum, establishing learner preferences is of paramount importance.

Results from our 26-item survey indicate that the majority of the 106 participants who responded to the survey felt that simulators are useful for the acquisition of procedural skills. There was perceived utility in simulation-based procedural education for the following medical procedures: central venous catheterization, thoracentesis, intubation, lumbar puncture and paracentesis. Participants favoured small-group teaching, led by either an attending or senior resident. Group sizes of no more than 3-4 learners for every simulator and no fewer than one instructor for every 3-4 learners were preferred. Duration of session of ≤ 3 hours was preferred. Participants preferred protected teaching time, early implementation of simulation-based procedural curriculum, with additional sessions offered throughout residency.

Participants felt that simulation-based educational sessions should cover primarily technical aspects of the procedure: use of equipment, review of anatomy, sterile techniques, troubleshooting techniques, and demonstration of technique by instructors. Participants felt that other cognitive or knowledge-based aspects of the procedure (review of indications, contraindications, complications, consent, procedural note, and review of current evidence on the procedure) could be taught by a variety of non-simulation-based teaching modalities. Participants' preferences for the use of simulators on technical aspects of the procedure are consistent with the use of simulators for experiential learning [22]. Instructors were felt to play an important role in simulation-based procedural education. Participants felt that the instructors should demonstrate techniques, observe learners, and provide feedback to learners. The majority of participants felt simulation would be useful for the acquisition of teaching skills. Lastly, commonly cited barriers to the implementation of simulation-based procedural education included limitations in time, realism of simulators, and resources.

Our results are consistent with current available evidence. For instance, participants favoured small group sessions. In a randomized trial examining the optimal teacher-to-learner ratio for suturing technique, optimal instructor-to-learner ratio was found to be 1 instructor for 4 students [23]. Small group size allows time for deliberate practice and feedback, elements previously demonstrated to be of educational value [10]. Secondly, participants valued protected teaching time and implementation of simulation sessions throughout residency. Integration of simulation teaching into a curriculum is an essential feature previously demonstrated to lead to effective learning [10]. Ongoing training throughout residency, rather than delivering simulation-based education as a one-time intervention may be particularly important for procedural skill retention [24]. Thirdly, the majority of participants (89%) felt that instructors should provide feedback. Consistently demonstrated in the literature is the value of feedback as an important feature of simulation-based education [10]. Fourthly, despite not having had prior exposure to incorporating a clinical scenario into procedural teaching, 78% of participants felt that this teaching technique would be useful. The Integrated Procedural Performance Instrument (IPPI), by integrating bench-top models to standardized patients in order to recreate clinical encounters, has been successfully used for assessment and teaching purposes [25,26].

Our study has several limitations. It was conducted at a single institution. However, we were able to achieve a high response rate of 83% and therefore, were able to capture responses from most of our trainees. Despite a high response rate, there remains a possibility of a response bias being present. The majority of participants had been previously exposed to simulator training and had generally high satisfaction with simulators, based on our previous experience [4]. Our survey results therefore may not be generalizable to learners with no prior exposure or those with predominantly negative prior experience with simulators. How the quality and amount of baseline experience with simulators influence learners' opinion is beyond the scope of our current study but does deserve further study. Secondly, because we were evaluating opinions from our learners, we were unable to evaluate whether incorporating trainees' stated preferences will necessarily lead to a more effective curriculum. In addition, preferences stated by the trainees may simply be a reflection of what simulator experience they have had to date and not based on comparing different educational experiences. A variety of simulator experiences were possible for our trainees, ranging from one-on-one teaching experience to one instructor per 6-8 trainees. We did not seek information about the specific type of experience our trainees may have had prior to completing the survey. Within the confines of our survey, we were unable to further explore reasons for our participants' stated preferences. Third, although our survey was constructed based on results from a literature scan on Pubmed using the following MeSH terms: *simulation; education; learning; teaching; and teach$*, we did not conduct a systematic review and therefore may have omitted important domains.

To our knowledge, our study provides a detailed examination of the learner's perspective on simulation-based procedural education, covering domains of interest to educators, including educational format and content. Our survey was rigorously developed [27] and had a high response rate. Our study indicates that learners are in favour of small-group simulation-based educational sessions for the acquisition of procedural skills in a number of procedures. Our participants assert that teaching sessions should be of short-duration, introduced early in residency with additional sessions throughout residency. Instructors should focus the use of simulation on the technical aspects of the procedures and provide feedback. These learner preferences should be considered in the development of simulation-based procedural curriculum.

Lastly, barriers to the implementation of simulation-based procedural education should be addressed. More recently, program requirements for Internal Medicine from the Accreditation Council for Graduate Medical Education mandate that institutions provide residents with access to training using simulation [13]. Residency training programs, therefore, should ensure adequate protected time in the curriculum and an adequate supply of procedural teachers.

## Conclusion

Our results suggest that residents value simulator-based procedural teaching in the form of small-group sessions. Protected teaching time, implementation of teaching early in residency, with additional teaching session throughout residency are favoured by participants. Implementation of a procedural curriculum may benefit both from taking learners' preferences into consideration and from available empirical evidence.

## Competing interests

The authors declare that they have no competing interests.

## Authors' contributions

DS: participated in research design, acquired, analyzed and interpreted data; drafted the paper. RYW: participated in research design and revised the paper critically. JMR: participated in research design, assisted in the acquisition of data, and revised the paper critically. PN: participated in research design and revised the paper critically. IWYM: conceived of the study, participated in research design, acquired, analyzed and interpreted data, drafted the paper, and revised the paper critically. All authors read and approved the final manuscript.

## Pre-publication history

The pre-publication history for this paper can be accessed here:

http://www.biomedcentral.com/1472-6920/10/77/prepub

## Supplementary Material

Additional file 1 **Appendix A - Survey Administered to Participants**. Survey items administered to participants. Click here for file
