# Supplementary material for: Use of simulator-based medical procedural curriculum: the learner's perspectives
Source: BMC Med Educ. 2010 Nov 8;10:77. doi: 10.1186/1472-6920-10-77 (PMC2988805; doi:10.1186/1472-6920-10-77)
Supplement: Additional file 1 — Appendix A - Survey Administered to Participants. Survey items administered to participants. [file 1472-6920-10-77-S1.DOC]

**Appendix A. Survey Administered to Participants**

1. Please indicate your gender:

Male  Female

1. Level of training

PGY 1  PGY2  PGY 3 and up

1. In the course of your training, have you had any exposure to medical simulators for procedural teaching? (Formal training refers to a course using simulators to teach procedural skills).

Yes, teaching sessions (i.e. with formal training)

Yes, practice sessions only (i.e. without formal training)

No, I have not had any exposure to medical simulators

1. If you have received formal training on medical simulators, please indicate for which procedures. (Check all that apply)

|  | Prior to residency | During residency |
| --- | --- | --- |
| Central line insertions |  |  |
| Lumbar punctures |  |  |
| ABG |  |  |
| Thoracentesis |  |  |
| Paracentesis |  |  |
| Arthrocentesis |  |  |
| Intubation |  |  |

1. If you have had practice sessions on simulators (i.e. with no formal training), please indicate for which procedures. (Check all that apply)

|  | Prior to residency | During residency |
| --- | --- | --- |
| Central line insertions |  |  |
| Lumbar punctures |  |  |
| ABG |  |  |
| Thoracentesis |  |  |
| Paracentesis |  |  |
| Arthrocentesis |  |  |
| Intubation |  |  |

1. Medical simulators are useful for…

|  | Strongly  Disagree | Somewhat Disagree | Neither Agree Nor Disagree | Somewhat Agree | Strongly Agree |
| --- | --- | --- | --- | --- | --- |
| Acquisition of procedural skills |  |  |  |  |  |
| Refining procedural skills |  |  |  |  |  |
| Assessment of procedural skills |  |  |  |  |  |
| Learning teaching skills |  |  |  |  |  |

1. Please rate the usefulness of incorporating a clinical scenario into procedural teaching using simulators.

Not at all useful

Somewhat not useful

Neither useful not no useful

Somewhat useful

Very useful

1. The following concepts relating to medical procedures should be taught by which modality?

|  | Simulation | Didactic lecture | Web-based module | Other means |
| --- | --- | --- | --- | --- |
| Review of evidence (e.g. content of JAMA series) |  |  |  |  |
| Indications/contraindications |  |  |  |  |
| Assessment of procedural skills |  |  |  |  |
| Review of equipment |  |  |  |  |
| Sterile techniques |  |  |  |  |
| Anatomy |  |  |  |  |
| Use of ultrasound |  |  |  |  |
| Diagnosis of complications |  |  |  |  |
| Treatment of complications |  |  |  |  |
| Troubleshooting techniques |  |  |  |  |
| Writing a procedural note |  |  |  |  |
| Obtaining informed consent |  |  |  |  |

Other means (please specify):

1. An introductory course on the following techniques would be useful during residency.

|  | Strongly  Disagree | Somewhat Disagree | Neither Agree Nor Disagree | Somewhat Agree | Strongly Agree |
| --- | --- | --- | --- | --- | --- |
| Strerile technique (gowning, gloving, sterile field, etc) |  |  |  |  |  |
| Hand-ties |  |  |  |  |  |
| Injection techniques for local anaesthetic |  |  |  |  |  |

1. If this was day one of my internal medicine residency, I would be interested in attending the following procedural training sessions on simulators:

|  | No | Maybe | Yes |
| --- | --- | --- | --- |
| Central line insertions |  |  |  |
| Ultrasound guided central line insertion |  |  |  |
| Lumbar punctures |  |  |  |
| ABG |  |  |  |
| Thoracentesis |  |  |  |
| Paracentesis |  |  |  |
| Intubation |  |  |  |
| Peripheral IV |  |  |  |
| PICC line insertion (peripherally inserted central catheter) |  |  |  |
| Introductory course (see question 9 above) |  |  |  |

1. Ideally simulator courses for medical procedures should be offered…

Most sessions at the start of residency

At the start of residency, with additional sessions throughout residency

Later in residency (PGY2/PGY3)

Timing of the courses is not important

No opinion

1. Ideally simulator courses for medical procedures should be scheduled

During academic half day

During CTU rotation (clinical teaching unit)

During ICU rotation (intensive care unit)

1. Simulator courses for medical procedures should be…

A mandatory part of the residency curriculum

An elective part of the residency curriculum

Some sessions should be mandatory, others elective

No opinion

1. Which sessions should be mandatory vs elective?

|  | Mandatory | Elective | Should not be offered | No opinion |
| --- | --- | --- | --- | --- |
| Central line insertion |  |  |  |  |
| Ultrasound-guided central line insertion |  |  |  |  |
| Lumbar puncture |  |  |  |  |
| ABG |  |  |  |  |
| Thoracentesis |  |  |  |  |
| Paracentesis |  |  |  |  |
| Intubation |  |  |  |  |

1. The maximum acceptable number of learners per simulator is:

no more than 1-2 learners per simulator

no more than 3-4 learners per simulator

no more than 5-6 learners per simulator

> 6 learners per simulator is acceptable

1. The minimum acceptable instructor to learner ratio is:

no fewer than 1 instructor per 1-2 learners

no fewer than 1 instructor per 3-4 learners

no fewer than 1 instructor per 5-6 learners

1 instructor for > 6 learners is acceptable

1. The optimal duration of a simulator session is…

1 hr

2 hrs

3 hrs

> 3 hrs

Full day session (with breaks)

1. The role of a supervisor in a simulation-based procedural education session is superfluous.

Strongly disagree

Somewhat disagree

Neither agree nor disagree

Somewhat agree

Strongly agree

1. Training session times should be held…

|  | Strongly  Disagree | Somewhat Disagree | Neither Agree Nor Disagree | Somewhat Agree | Strongly Agree |
| --- | --- | --- | --- | --- | --- |
| At specified, protected times |  |  |  |  |  |
| At specified, unprotected times |  |  |  |  |  |
| Simulators should be freely available |  |  |  |  |  |

1. Please rank your preference for ideal locations of medical simulators.

|  | Least preferred | 4^th^ most preferred | 3^rd^ most preferred | 2^nd^ most preferred | Most preferred |
| --- | --- | --- | --- | --- | --- |
| Center away from the hospital |  |  |  |  |  |
| Center within the hospital |  |  |  |  |  |
| Directly on the ward |  |  |  |  |  |
| In the doctor’s lounge |  |  |  |  |  |

Other (Please specify):

1. Medical simulator sessions should be taught by:

|  | Strongly  Disagree | Somewhat Disagree | Neither Agree Nor Disagree | Somewhat Agree | Strongly Agree |
| --- | --- | --- | --- | --- | --- |
| An attending physician |  |  |  |  |  |
| A senior resident (or fellow) |  |  |  |  |  |
| A trained technician |  |  |  |  |  |

1. The instructor should:

|  | Strongly  Disagree | Somewhat Disagree | Neither Agree Nor Disagree | Somewhat Agree | Strongly Agree |
| --- | --- | --- | --- | --- | --- |
| Demonstrate technique |  |  |  |  |  |
| Observe my procedure |  |  |  |  |  |
| Teach evidence behind procedural steps |  |  |  |  |  |
| Provide feedback |  |  |  |  |  |

1. Useful feedback from the instructor includes (please check all that apply):

Written feedback

Oral feedback

Video review of my performance with the instructor

No feedback should be provided

1. In general, simulators do NOT help learners acquire procedural skills.

Strongly disagree (they DO help)

Somewhat disagree

Neither agree nor disagree

Somewhat agree

Strongly agree (they DON’T help)

1. Please describe any perceived barriers to the use of medical simulators for procedural teaching.
2. Any additional comments?
